# Effect of Energy Utilization and Economic Growth on the Ecological Environment in the Yellow River Basin

**DOI:** 10.3390/ijerph20032345

**Published:** 2023-01-28

**Authors:** Chenyu Lu, Wei Liu, Ping Huang, Yueju Wang, Xianglong Tang

**Affiliations:** 1 School of Architecture and Urban Planning, Lanzhou Jiaotong University, Lanzhou 730070, China; 2 College of Geography and Environmental Science, Northwest Normal University, Lanzhou 730070, China; 3 Yulin Huadong Middle School, Yulin 719000, China

**Keywords:** energy, economy, environment, Yellow River basin, impulse response

## Abstract

In the 21st century, problems relating to energy, economy, and the environment have become increasingly severe across the world, and critical issues around environmental pollution, ecological imbalance, and an energy crisis have emerged. The Yellow River basin is an important ecological barrier, economic region, and energy base in Northern China. Environmental pollution in the Yellow River basin has become increasingly problematic, especially since the reform and opening up of China, along with the rapid development of the industrial economy and mining for energy resources. In this study, 64 of the 73 prefecture-level cities in the Yellow River basin were selected as the research object, including 18 cities in the downstream region, 26 cities in the midstream region, and 20 cities in the upstream region. The data used in this study were from 2004 to 2019. On the basis of temporal variation and spatial differentiation of the three factors of economy, energy, and environment, the impulse response function and the generalized method of moments (GMM) were adopted to evaluate the effects of energy utilization and economic growth on the ecological environment. Their roles in affecting the ecological environment were analyzed along with the underlying mechanisms. Overall, energy utilization, economic growth, and ecological environment are in good condition, showing a steady upward trend. Regional differences still exist, but the gap is gradually narrowing. There are some differences in the impulse response of the ecological environment to the economic growth and energy utilization in the upstream, midstream, and downstream regions of the Yellow River basin. The effect is leveled out or weakened in the middle and later phases of the impact. Compared with the downstream and upstream regions, economic growth and energy utilization in the midstream regions have less impact on the ecological environment. The two factors of energy utilization potential and economic potential have significant positive impacts on the ecological environment. The current situation of energy utilization has to some extent a positive impact on the ecological environment. Economic scale has a certain negative impact on the ecological environment.

## 1. Introduction

Energy is crucial for economic growth and societal development for all nations. The rational utilization of energy is related to the ecological environment and ability of a country and a region, as well as the level of social and economic development. Due to the huge difference in energy endowment in different regions, there is always a certain contradiction between energy supply and demand and the development of local economy, which has a restricting effect on the development of local economy. At the same time, rapid economic growth is inevitably accompanied by a large amount of energy consumption, which has brought a series of ecological and environmental protection problems. As the carrier of energy and economy, the environment plays a dual role in promoting and hindering the development of energy and economy. In the 21st century, problems relating to energy, economy, and the environment have become increasingly severe across the world, and critical issues around environmental pollution, ecological imbalance, and energy utilization have emerged. In this context, various countries and regions in the world have paid attention to the relationship between energy utilization and economic growth on ecological environment, carrying out extensive research. The Yellow River Basin is an ecological corridor connecting the Qinghai–Tibet Plateau, the Loess Plateau, and the North China Plain, an important economic corridor for the development of the Belt and Road Initiative, and an important link covering and radiating the economic and social development of the eastern, central, and western provinces. Maintaining the health of the Yellow River plays an important role in the national economic and social development and ecological security [1]. For a long period, the economic growth and development mode of the Yellow River basin have mainly relied on mining energy resources and agricultural production. The extensive mode of development has imposed a severe challenge to the fragile ecological environment and constantly threatened sustainable development in the Yellow River basin [2,3]. Environmental pollution in the Yellow River basin has become increasingly problematic, especially since the reform and opening up of China, along with the rapid development of the industrial economy and mining for energy resources. Increasing pollution has gradually limited the growth of the national economy. In 2015, the United Nations put forward 17 sustainable development goals [4], covering all aspects of economy, environment, and society. In recent years, China has conducted various discussions and debates on the utilization of natural resources, protection of the ecological environment, and improvements in the Yellow River basin, and a strategy of promoting high-quality development has been proposed. Currently, protection of the ecological environment and high-quality development of the Yellow River basin have become a part of China’s national key strategy. Thus, it is of great significance to achieve sustainable development of energy, the economy, and the environment in the Yellow River basin and remove the dependence on the conventional mode of development. This is also an inevitable requirement of regional transformation and development in the Yellow River basin, together with the implementation of the national key strategy.

**Figure 1:**
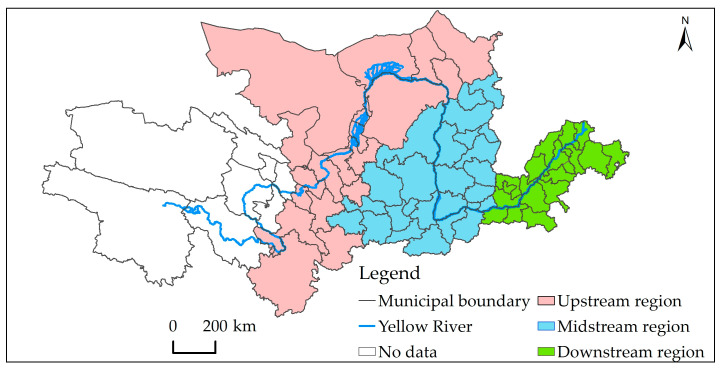
Schematic diagram of the research area.

In this study, 64 of the 73 prefecture-level cities in the Yellow River basin (Figure 1) were selected as the research object, including 18 cities in the downstream region, 26 cities in the midstream region, and 20 cities in the upstream region. The data used in this study were from 2004 to 2019. On the basis of temporal variation and spatial differentiation of the three factors of economy, energy, and environment, the impulse response function and the generalized method of moments (GMM) were adopted to evaluate the effects of energy utilization and economic growth on the ecological environment. Their roles in affecting the ecological environment were analyzed along with the underlying mechanisms. In doing so, on one hand, this study can supplement and improve the research systems of environmental and energy economics, enriching the theory relating to human geography and sustainable development; on the other hand, it can provide a scientific basis for decision making with regard to energy utilization, ecological environment protection, and high-quality development in the Yellow River basin, as well as promote the smooth implementation of sustainable development strategy, thus having important practical value.

The study aimed to address three research questions. First, we measured the temporal changes and spatial differentiation of energy utilization, economic growth, and ecological environment. Second, we analyzed the impact response of energy utilization and economic growth on ecological environment. Third, we explored the mechanism of energy utilization and economic growth’s impact on the ecological environment.

## 2. Literature Review

Globally, researchers have conducted extensive studies into the relationships among energy, economy, and the environment. Initially, this research tended to focus on binary interactions between these factors. The environmental Kuznets curve (EKC) is generally applied to explore the relationships between environment and economy. For example, Ahmad et al. conducted a study into the inverted U-shaped relationship between real gross domestic product (GDP) per capita and carbon dioxide emissions per capita [5]. Arnaut et al. verified that the EKC could be adopted in Greenland [6]. Murshed et al. confirmed the validity of the EKC hypothesis based on data from five South Asian economies [7]. Uddin et al. examined the causal relationship between economic growth and environmental degradation for 115 countries, and found mixed support for the environmental Kuznets curve (EKC) hypothesis, confirming the U-shaped EKC for all countries in terms of CO_2_ and an inverted U-shaped EKC in terms of both CH_4_ and PM_2.5_ emissions for the low-, low–middle-, and high-income countries [8]. For the interrelationship between energy and economy, previous studies mostly used the Granger causality test and co-integration theory. Asiedu et al. found a two-way causal relationship between economic growth and renewable energy in 26 developed countries in Europe [9]. Kasperowicz et al. developed the panel co-integration test and other methods and found that, in 29 European countries, a long-term balance exists between economic growth and renewable energy consumption [10]. Luqman et al. investigated asymmetric effects of renewable and nuclear energy on economic growth by extending the production function along with labor and capital for Pakistan’s economy [11]. Emir et al. examined the relationship among energy intensity, carbon emissions, renewable energy consumption, and economic growth for the case of Romania given the conflicting evidences in the literature between 1990 and 2014 on a quarterly basis, and they showed feedback causality between energy intensity and economic growth, while unidirectional causality was seen from renewable energy consumption to economic growth [12]. Koçak et al. explored the relationship between renewable energy consumption and economic growth for the period of 1990–2012 in nine Black Sea and Balkan countries, revealing a long-term balance relationship between renewable energy consumption and economic growth, while renewable energy consumption had a positive impact on economic growth [13]. Since the 1980s, to solve the contradiction among energy, environment, and economic development [14], researchers have paid attention to the relationships among economy, energy, and environment, and the energy–economy–environment (3E) model has been established for research in this field. Magazzino conducted an empirical study in Italy and reported that carbon dioxide emissions, economic growth, and energy consumption were all in two-way causal relationships [15]. Zaman et al. studied the interactions among energy consumption, economic growth, and carbon dioxide emissions [16]. Hanley et al. found that improvements in energy efficiency could significantly promote economic development but worsen environmental quality [17]. Lenzen et al. studied the relationships among elements of the 3E system in Australia [18]. Bastola et al. examined the causal relationships among energy consumption, pollution emission, and economic growth for Nepal, employing time series econometric methodology [19].

In China, research into the relationships among energy, economy and, environment began in the 1990s and was generally consistent with research trends across the world. In recent years, China has mainly focused on the development of 3E systems in energy, environment, and economy, especially on the simultaneous cooperative analysis of 3E systems. Li et al. made a comprehensive analysis of the coordinated development and spatial patterns of China’s provincial 3E system [20]. Chen et al. calculated the level of coordination of 3E systems in the Yangtze River Delta region [21]. Lu et al. analyzed the dynamic evolution characteristics of the 3E system in Shandong Province [22]. By analyzing the coupling mechanism of systems and conducting the coupling model of energy, economy, and environment (3E), Lu et al. estimated the coupling degree of 3E and analyzed the characteristics of coupling changes and spatial difference of four regions [23]. Li et al. used various mathematical analysis methods and GIS technology in Gansu Province to conduct a spatiotemporal comprehensive measurement of coordination and sustainable development of a population–economy–society–resource–environment system [24]. Xu et al. analyzed the coupling relationship of the 3E system based on big data [25]. Chai et al. proposed a quantitative analysis framework based on the water–energy–food–economy–society–environment nexus, and the causality relations among water, energy, food, economy, society, and environment were studied and quantified [26].

In general, the research conducted has made great progress, but a few shortcomings still exist. First, from the research perspective, the factors of energy, economy, and the environment are incorporated into a unified framework. As with the relationships among the three factors, there has been very little quantitative research into the effect of energy utilization and economic growth on the ecological environment. The relevant studies are still in the preliminary exploration stage. Second, comprehensive quantitative research into the dynamic effects and mechanisms among energy utilization, economic growth, and the ecological environment in a systematic manner using impulse response function and GMM based on a vector auto-regression (VAR) model has rarely been carried out, which should be further analyzed. Third, there have been very few systematic and comprehensive comparative studies into prefecture-level cities in the Yellow River basin in China. With this study, we aimed to address this knowledge gap.

## 3. Data and Methods

### 3.1. Index System and Data Sources

On the basis of the results and conclusions of the existing research [27,28,29,30,31], taking the principles of scientificity, representativeness, accessibility, and integrity into consideration, and combining the results with the actual regional situation, an energy utilization, economic growth, and ecological environment measurement index system for the Yellow River basin was constructed (Table 1), which is original to this study and different from the relevant index system in the existing literature. The timespan of the research was from 2004 to 2019 and comprised data from prefecture-level cities in the Yellow River basin (the cities in Qinghai Province were not included due to a lack of data). The data originated from regional statistical yearbooks, environmental yearbooks, energy yearbooks, national economic and social development statistical bulletins, environmental bulletins, and other relevant statistics and literature sources. Vector data at a prefecture-level city scale in the Yellow River basin were obtained from the Resources and Environment Science Data Center of the Chinese Academy of Sciences and extracted using ArcGIS.

### 3.2. Research Methods

#### 3.2.1. The VAR Model

The VAR model was first formally proposed by Christopher Sims in 1980 [32]. A VAR model was constructed using an unstructured method, to facilitate the study of interrelationships among variables. The mathematical expression of the VAR model is as follows:

(1) $$y_{t}=A_{1}y_{t-1}+\cdots +A_{p}y_{t-p}+{Bx}_{t}+\varepsilon_{t},$$

where $y_{t}$ is the *k-th* dimensional endogenous variable vector, $x_{t}$ is the *d-th* dimensional exogenous variable vector, *t* = 1, 2, …, *T* (*T* is the number of samples), *P* is the lag order, *A* and *B* are the coefficient matrices, and $\varepsilon_{t}$ is the disturbance term.

#### 3.2.2. Impulse Response Function

The impulse response function is used to quantify the dynamic effect on a system when it is impacted [33]. Stationarity and co-integration tests should be performed before the impulse response function analysis [34,35]. For the stationarity test, an ADF (augmented Dickey–Fuller) test was used to judge the stationarity of the time series of each variable based on the logarithmization of the time series data. For the co-integration relationship test, a judgment of the co-integration relationship among the multivariate variables was required. Thus, the Johansen co-integration test was used to measure whether a long-term equilibrium relationship existed among the variables.

The expression for the impulse response function is as follows [36]:

(2) $$y_{t}=c_{ij}^{0}\varepsilon_{t}+c_{ij}^{1}\varepsilon_{t-1}+c_{ij}^{2}\varepsilon_{t-2}+\cdots +c_{ij}^{q}\varepsilon_{t-j},$$

where the corresponding function of *y_i_* caused by the impulse is $c_{ij}^{0}$, $c_{ij}^{1}$, $c_{ij}^{2}$, …, and $c_{ij}^{q}$. In the equation, $c_{ij}^{q}=\frac{\partial yt+q}{\partial \varepsilon t}$ indicates, in time period *t*, the effect of the increase in the disturbance term of the *j-th* variable by one unit on the value of the *i-th* variable of period *t* + *q* when the disturbance in the other periods is constant. Lastly, the sum of the *j-th* perturbation term ε_i_ on the value of *y_i_* in all periods can be calculated from Equation (2).

#### 3.2.3. GMM Model

The GMM, first proposed by Hansen [37], is a parameter moment estimation method based on some moment conditions being satisfied by the actual parameters of the model. The idea of GMM is to estimate the model parameters using the sample moment condition and select the smallest value of estimated distance [38].

When the ecological environment was used as the explained variable, and energy utilization and economic growth were used as the explanatory variables, the regression model could be expressed as follows:

(3) $${lnX}_{it}=\beta_{0}+\beta_{1}{IS}_{i}+\beta_{2}{TI}_{i}+\varepsilon_{i},$$

where *X* is the ecological environment, *IS* is the energy consumption, *TI* is the economic growth, and $\varepsilon_{i}$ is the error term.

## 4. Results and Discussion

### 4.1. Variation in Energy Utilization, Economic Growth, and Ecological Environment over Time

Four typical years (2004, 2008, 2013, and 2019) were selected to produce a kernel diagram of the three major systems of energy utilization, economic growth, and the ecological environment, using kernel density estimation.

**Figure 2:**
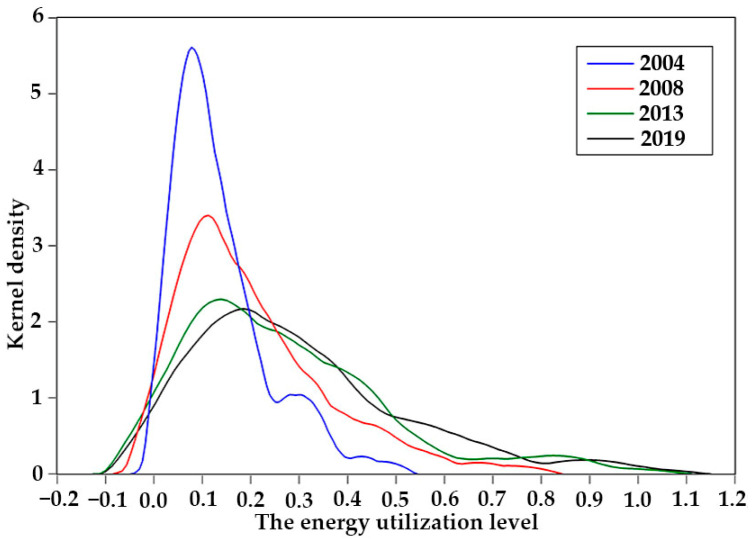
Kernel density distribution of the energy utilization system.

**Figure 3:**
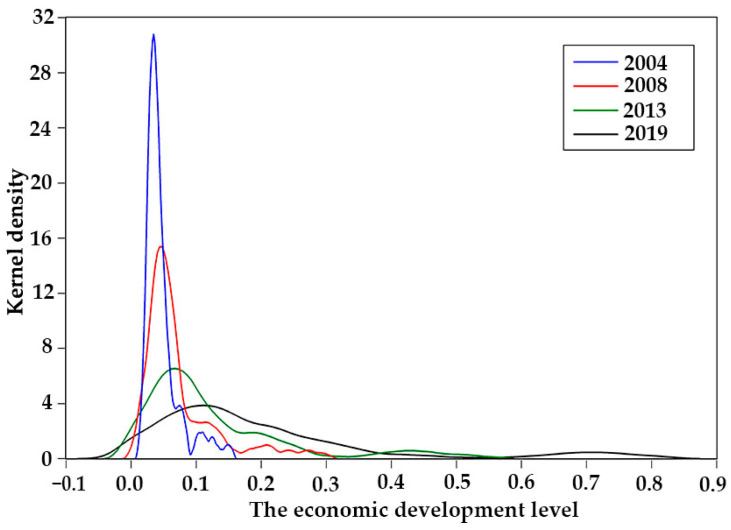
Kernel density distribution of the economic growth system.

**Figure 4:**
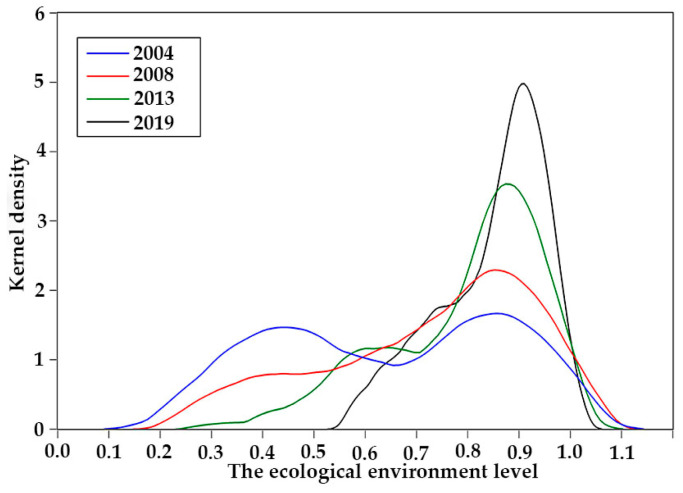
Kernel density distribution of the ecological environment system.

In terms of energy utilization (Figure 2), the function center showed a right-shifting trend over the years. The magnitude of the right shift was roughly the same in each year, indicating that the improvement in energy utilization level was relatively stable. The wave peaks in 2004 and 2008 were relatively high, while the peaks in 2013 and 2019 were relatively low, indicating that the gap in the level of regional energy utilization was gradually narrowing. In terms of economic growth (Figure 3), the function center also showed a trend of right shift over the years, and the right shift range was roughly equal, indicating that the level of economic development was steadily improving year by year. In 2004 and 2008, the peaks were relatively high, while, in 2013 and 2019, the peaks were relatively low. The peaks were all in the single peak style for the four years, indicating that the gap between the economic development level was gradually narrowing. In terms of the ecological environment (Figure 4), the function center developed significantly on the right side, indicating that the overall level of ecological and environmental protection was being significantly improved. The shape of the wave peak became more and more steep, and the peak width showed a trend of gradually narrowing, indicating that there was a gap in the level of ecological environment across the region. However, the gap was gradually narrowing.

### 4.2. Spatial Variation in Energy Utilization, Economic Growth, and the Ecological Environment

**Figure 5:**
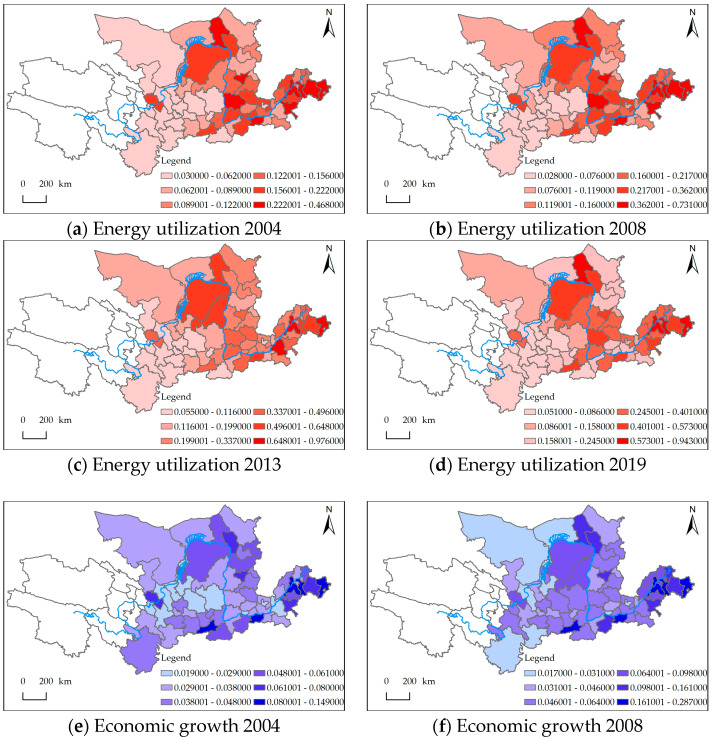

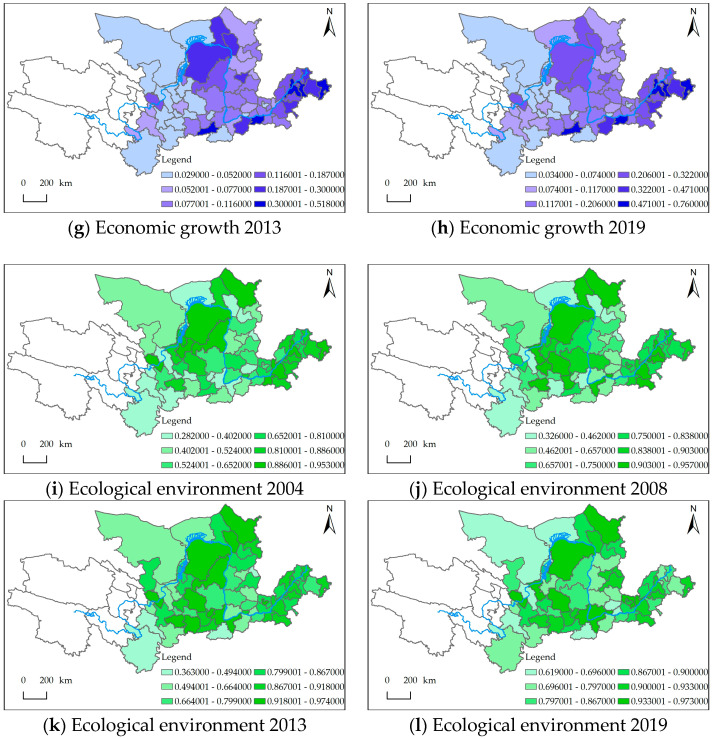
Spatial distribution of energy utilization, economic growth, and the ecological environment.

The spatial distributions of the energy utilization, economic growth, and ecological environment systems were obtained for the four typical years of 2004, 2008, 2013, and 2019 (Figure 5). The overall development trend of energy utilization was good, and the level of energy utilization in various regions improved, but regional differences remained. In the early years, the energy utilization status of regional central cities and coastal cities was good. After that, the energy utilization level in areas with rich resources and a good industrial foundation significantly improved. More recently, the overall energy utilization situation in the various regions was good, but some regions still had a low level of energy utilization. As with economic growth, the overall situations of the cities showed an upward trend, but there were some regional differences. In the early stage, the downstream coastal cities and some regional central cities with a good industrial foundation in the midstream regions showed rapid economic development momentum and obvious economic growth. After that, the cities with geographical advantages and rich resources in the midstream and upstream regions also gradually improved. More recently, the economic development in the downstream region was still the best. As with the ecological environment, the overall development trend was also good. The level of the ecological environment improved over the years, and the regional gap was significantly narrowed. In the early stage, the ecological environment level of the “Ji-characters” region of the Yellow River basin and the downstream region was relatively better than that of the other regions. Later, the ecological environment levels in the midstream and downstream regions were better than those in the upstream regions. More recently, the level of the ecological environment rapidly improved.

### 4.3. Impulse Response of the Ecological Environment to Energy Utilization and Economic Growth

We next conducted logarithmic processing of relevant variables, followed by the stationarity test (ADF test), to further develop the VAR model. The Johansen co-integration test was used to determine whether a long-term equilibrium relationship existed among the variables. Then, the impulse response function was used to evaluate the dynamic effect of energy utilization and economic growth on the ecological environment.

#### 4.3.1. Upstream Regions

**Figure 6:**
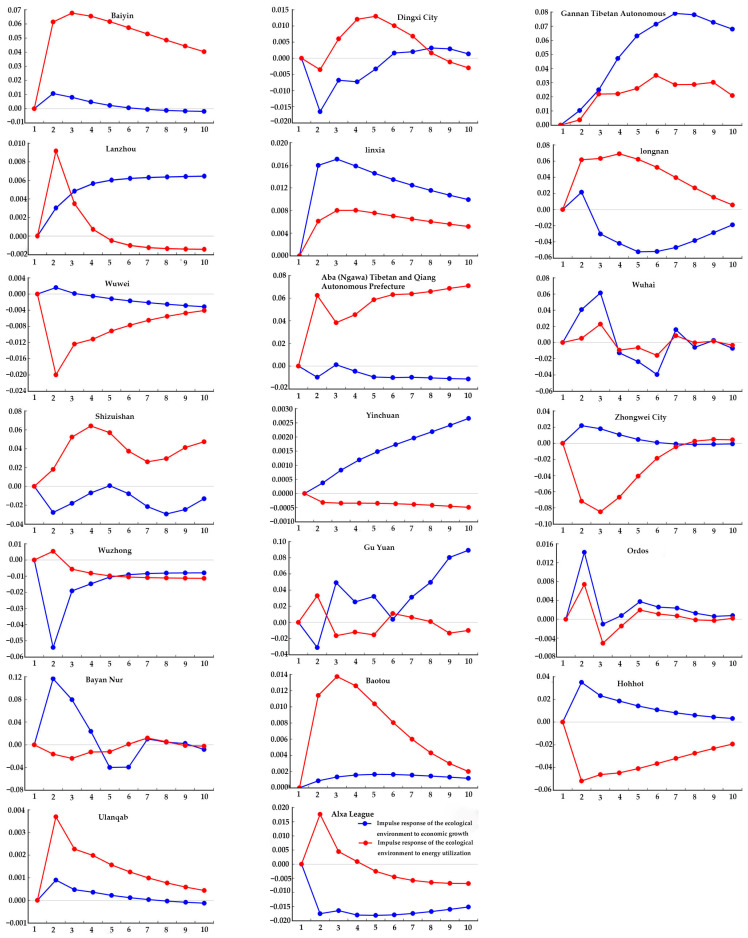
Impulse response of the ecological environment to energy utilization and economic growth in the upstream area.

As shown in Figure 6, for the upstream regions, the overall effect of energy utilization on the ecological environment was greater than that of economic growth. The response was more obvious in the middle stage of the impact. At first, the impact of energy utilization on the ecological environment was more obvious. With the gradual increase in the environmental protection and improvement of the ecological environment, the impact on the ecological environment also gradually weakened and became stable at last. The improvement of economic level also accelerated energy consumption and further affected the quality of the ecological environment; however, this was still weaker than the impact of energy utilization. In the cities of Baiyin, Dingxi, Shizuishan, Baotou, and Ulanqab, during the entire response period, the effect of energy utilization on the ecological environment was significantly greater than that of economic growth. For the impact of energy utilization on the ecological environment, the cities of Lanzhou, Wuzhong, and Ordos responded significantly in the initial stage, while the peak value of 0.0085 was obtained in Lanzhou in Phase 2. In the cities of Dingxi, Longnan, Baotou, and Shizuishan, the impact was significant in the middle stage, with a peak value of 0.0613 seen in Shizuishan in Phase 4. The city of Aba (Ngawa) Tibetan and Qiang Autonomous Prefect showed a peak value of 0.0827 in Phase 9, while Wuhai, Yinchuan, Wuzhong, and Bayan Nur responded weakly.

#### 4.3.2. Midstream Regions

**Figure 7:**
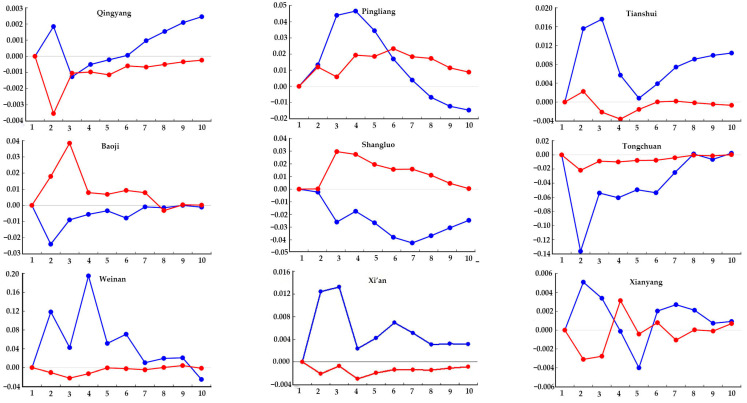

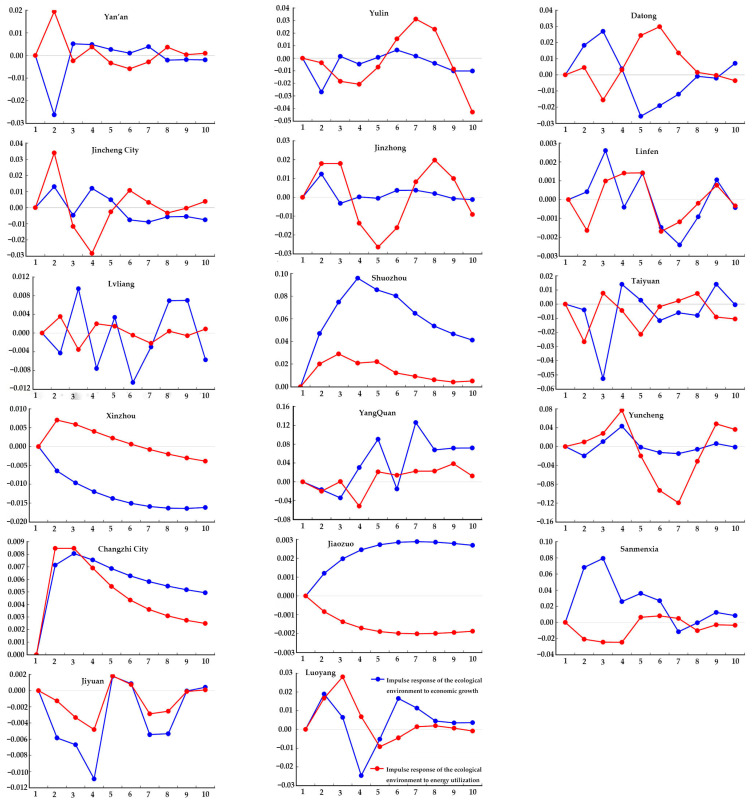
Impulse response of the ecological environment to energy utilization and economic growth in the midstream regions.

As shown in Figure 7, for the midstream regions, the impact of economic growth and energy utilization on the ecological environment was roughly the same on the whole region. The impact effect was relatively obvious in the initial and medium stages. Compared with the downstream and upstream regions, economic growth and energy utilization in the midstream regions had less impact on the ecological environment. The impact of economic growth and energy utilization on the ecological environment in Xianyang, Datong, Lvliang, Taiyuan, and other places was symmetrically distributed. There was a simultaneous impact of economic growth and energy utilization on the ecological environment, but the impact was gradually reduced. In the cities of Sanmenxia, Linfen, and other places, a negative response to the impact of energy utilization was observed in the initial stage, while a positive response to the impact of economic growth was obtained. As time went on, their impact on the ecological environment tended to be stable. Baoji and Yan’an showed a positive response to the impact of energy utilization in the initial stage and a negative response to the impact of economic growth. As time went on, their impact on the ecological environment tended to become stable. The impact of economic growth on the ecological environment in the cities of Xi’an, Shuozhou, and Weinan was higher than the impact of energy utilization on the ecological environment. As time went on, the impact of the two factors on the ecological environment gradually decreased and tended to become stable.

#### 4.3.3. Downstream Regions

**Figure 8:**
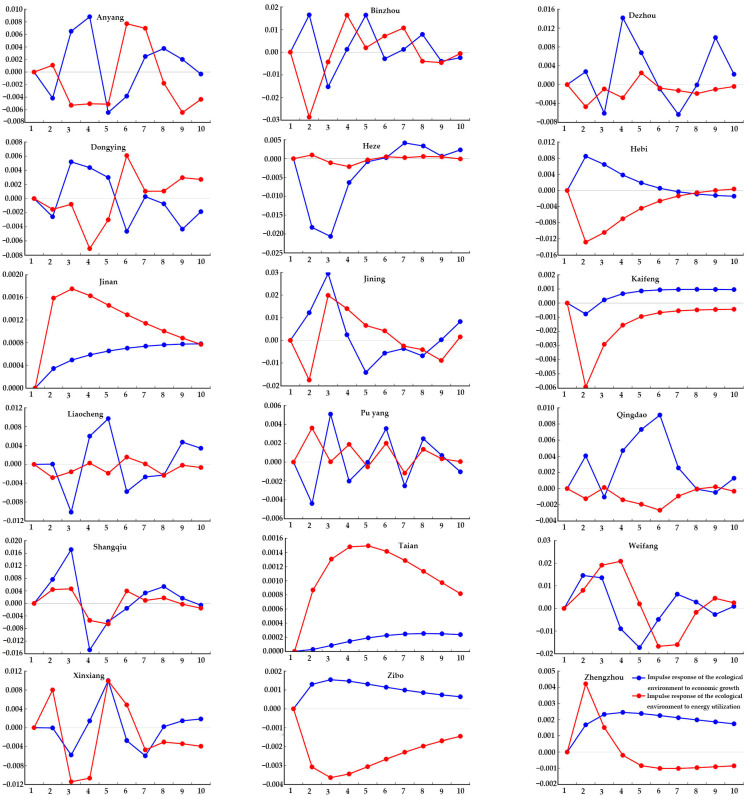
Impulse response of the ecological environment to energy utilization and economic growth in the downstream regions.

As shown in Figure 8, for the downstream regions, the overall impact of economic growth on the ecological environment was greater than that of energy utilization. The impact was relatively obvious in the early and middle stages. In the early stage, with the rapid economic growth, the ecological environment suffered a certain degree of pollution, and the environmental pressure was great. Later, with the increased investment in environmental protection, the quality of the ecological environment gradually improved. The impact gradually decreased in the middle stage. The economic growth in Qingdao and Zhengzhou had less impact on the ecological environment, and the impact gradually eased off at the end of the research (2019). The impact of economic growth in Dezhou and Liaocheng on the ecological environment was clearly greater than the impact of energy utilization. In particular, Dezhou reached a peak value of 0.0149 in Phase 4. The impact of economic growth in Binzhou and Pu yang on the ecological environment was roughly the same as the impact of energy utilization.

### 4.4. The Effect of Energy Utilization and Economic Growth on the Ecological Environment

The GMM was used to evaluate the influence of energy utilization (utilization status *X*_1_ and utilization potential *X*_2_) and economic growth (economic scale *X*_3_ and economic potential *X*_4_) on the ecological environment (STHJ(−1)). The results are shown in Table 2.

A variation of one unit in energy utilization status would cause a variation of 0.1768 units in the ecological environment. As manufacturing technology has progressed, newer, cleaner energy has been widely applied. As a result, the energy utilization efficiency has been improved, and the adverse impact of energy consumption on the ecological environment has been gradually weakened. This can reduce the pollution and damage to the ecological environment to a certain extent. A variation of one unit in energy utilization potential would cause a variation of 1.7215 units in the ecological environment. As energy utilization efficiency has improved, the growth rate of energy consumption has become lower than the growth rate of the economy. With continuous economic growth, a virtuous cycle can be formed, and the negative impact on the ecological environment can ultimately be reduced. A variation of one unit in the economic scale would cause a variation of 0.0779 units in the ecological environment. In the early stage, economic development caused some pollution and damage to the ecological environment. With the deepening of economic transformation and the correct guidance of national policies, this phenomenon was effectively controlled. The negative impact on the ecological environment was maintained at a low level. A variation of one unit in economic potential would cause a variation of 1.3149 units in the ecological environment. In the process of economic development, along with the development of tertiary industry and the optimization of the industrial structure, the ecological environment would be significantly improved.

### 4.5. Discussion

In recent years, although researchers have carried out research into the 3E system, there is still relatively little published work. Zhao et al. found that levels of energy consumption, economic growth, and the ecological environment in China showed different growth trends and that there were spatial correlations among the three factors [39]. The differences among these three factors across different provinces have gradually narrowed. Li et al. put forward two key issues relating to energy production and socioeconomic development in the Yellow River basin [40]. Lin et al. used the SBM (super slacks-based model) super-efficiency model based on unexpected output and the Malmquist index to calculate and analyze the resources and environmental efficiency of cities above prefecture level in the Yellow River basin [41].

To achieve coordinated and sustainable development of energy utilization, economic growth, and the ecological environment, some measures and suggestions are proposed. Firstly, advanced technology should be introduced, while out-of-date production capacity and technologies should be eliminated. This could actively improve energy utilization efficiency. Clean energy should be widely used to continuously optimize the energy consumption structure. Secondly, each region should explore in depth its own comparative advantages and then develop regional economies with its own characteristics, to promote regional division of labor and cooperation. Thirdly, ecological and environmental protection should be strengthened in the adjustment of regional industry and its layout. For example, the regions rich in coal resources in the midstream and upstream regions of the Yellow River basin should promote the clean and efficient utilization of coal-based fossil energy. It is also important to increase the recycling of waste. Lastly, local governments should give proper policy guidance and guarantees according to the actual regional situation.

## 5. Conclusions

From the perspective of the spatial and temporal evolution of energy utilization, economic growth, and the ecological environment in the Yellow River basin, the overall development trend of energy utilization is good, and the level of energy utilization in the various regions has improved to varying degrees. Regional differences still exist, but the gap is gradually narrowing. Overall economic growth is also in good condition, showing a steady upward trend. There are certain regional differences in the economic development of various regions, but this gap is also gradually narrowing. The ecological environment has been significantly improved. There are some gaps in the different regions, but these gaps are gradually narrowing. This conclusion corresponds to the first research question, revealing the temporal changes and spatial differentiation of energy utilization, economic growth, and ecological environment.

In general, there are some differences in the impulse response of the ecological environment to the economic growth and energy utilization in the upstream, midstream, and downstream regions of the Yellow River basin. The effect has been leveled out or weakened in the middle and later phases of the impact. Compared with the downstream and upstream regions, economic growth and energy utilization in the midstream regions have less impact on the ecological environment. For the upstream regions, the overall impact of energy utilization on the ecological environment is greater than the impact of economic growth, and the effect is more distinct in the middle phases of the impact. For the midstream regions, the impact of economic growth and energy utilization on the ecological environment is roughly the same, and the impact is relatively obvious in the initial and middle stages. For the downstream regions, the overall impact of economic growth on the ecological environment is greater than the impact of energy utilization. The impact is relatively obvious in the early and middle stages. This conclusion corresponds to the second research question, revealing the impact response of energy utilization and economic growth on ecological environment.

The two factors of energy utilization potential and economic potential have significant positive impacts on the ecological environment. Improvements in energy utilization potential and economic potential can promote improvements in the quality of the ecological environment quality and have a significant, positive effect. The current situation of energy utilization has to some extent a positive impact on the ecological environment. With improvements in and optimization of the current energy utilization situation, it will be possible to promote the improvement of the quality of the ecological environment and show a certain positive effect. However, this positive effect is relatively weak. Economic scale has a certain negative impact on the ecological environment, and improvements in economic scale are not conducive to improvements in the quality of the ecological environment. It also has a relatively weak negative effect. The negative impact on the ecological environment is maintained at a low level. This conclusion corresponds to the third research question, revealing the mechanism of energy utilization and economic growth’s impact on the ecological environment.

This study had some limitations. Firstly, the data sources are very limited, and the indicators of non-fossil energy were missing or incomplete, which may have led to the insufficient control of variables in the status quo of energy utilization. Secondly, in the selection of ecological environmental indicators, environmental indicators were targeted on, whereas there was a relative lack of ecological indicators. Furthermore, this study primarily considered energy utilization status, energy utilization potential, economic scale, and economic potential as the influencing factors of ecological environment, while other influencing factors including population were not included in the construction of relevant indicators. These limitations can be improved in future research.

## Figures and Tables

**Table 1:** Energy utilization, economic growth, and ecological environment measurement index system.

Target Layer A	Target Layer B	Target Layer C
Energy utilization	Utilization status	Total energy consumption
	Utilization potential	Energy consumption per unit GDP
		Energy consumption elasticity coefficient
Economic growth	Economic scale	Gross industrial output value
		Added value of tertiary industry
		Total retail sales of social consumer goods
	Economic potential	Proportion of tertiary industry
		Economic density
Ecological condition	Environmental pollution	Industrial wastewater discharge volume
		Industrial sulfur dioxide emissions
		Industrial particulate matter emissions
	Pollution control and ecological improvement	Comprehensive utilization rate of industrial solid waste
		Standard reaching rate of industrial wastewater discharge

**Table 2:** GMM results of the impact of energy use and economic growth on the ecological environment.

Variable	Coefficient	Standard Error	T Statistics	Probability
STHJ (−1)	0.5321	0.0046	115.5510	0.0000
*X*_1_	0.1768	0.0107	16.4792	0.0000
*X*_2_	1.7215	0.0600	28.7017	0.0000
*X*_3_	−0.0779	0.0156	−5.0062	0.0000
*X*_4_	1.3149	0.0692	18.9924	0.0000

(−1): the order of lag 1.

## Data Availability

The data presented in this study are available on request from the corresponding author.
